# Neoadjuvant and Adjuvant Immunotherapy in Non-Small Cell Lung Cancer—Clinical Trials Experience

**DOI:** 10.3390/cancers13205048

**Published:** 2021-10-09

**Authors:** Izabela Chmielewska, Katarzyna Stencel, Ewa Kalinka, Rodryg Ramlau, Paweł Krawczyk

**Affiliations:** 1 Chair and Department of Pneumonology, Oncology and Allergology, Medical University of Lublin, 20-059 Lublin, Poland; krapa@poczta.onet.pl; 2 Chair and Department of Oncology, Poznan University of Medical Sciences, 61-701 Poznan, Poland; katarzyna.stencel@skpp.edu.pl (K.S.); rodryg.ramlau@skpp.edu.pl (R.R.); 3 Department of Chemotherapy, Clinical Hospital of Lord Transfiguration, 60-659 Poznan, Poland; 4 Department of Oncology, Polish Mother’s Memorial Hospital—Research Institute, 90-302 Lodz, Poland; ewakalinka@wp.pl

**Keywords:** early-stage, non-small cell lung cancer, neoadjuvant, adjuvant, immunotherapy

## Abstract

**Simple Summary:**

Surgical resection remains the gold standard of early-stage non-small cell lung cancer (NSCLC) treatment. However, only a minority of resected patients remain recurrence-free at 5 years. Systemic treatment with cisplatin-based chemotherapy after surgical resection has been shown to improve survival in this setting. In the last few years, immunotherapy has established its position in treatment of metastatic lung cancer patients. Can the phenomenal results of this treatment be directly transferred to early NSCLC patients? Clinical trials with immunotherapy in this indication are ongoing, some with already promising results. In order to immediately prove the efficacy of immunotherapy in preoperative use, the surrogates of overall and progression free survival have to be validated. In this article, we review the data in support of immunotherapy in adjuvant and neoadjuvant treatment of early NSCLC patients together with new definitions of primary end points of these studies.

**Abstract:**

Across all tumor types, we observe that the role of immunotherapy has increased rapidly. Due to a number of potential advantages, it is considered in neoadjuvant treatment of localized tumors. In neoadjuvant settings, immunotherapy addresses micrometastatic diseases at the moment of their formation. However, some issues concerning neoadjuvant and adjuvant immunotherapy still has to be covered. The choice of drug and use of monotherapy or combination regimens remains unclear. The timing of surgery and preoperative evaluation of neoadjuvant immunotherapy efficacy is challenging. Although there is currently limited confirmed clinical data to support the use of immune checkpoint blockade in the neoadjuvant and adjuvant settings, there are many studies exploring this strategy in NSCLC patients.

## 1. Introduction

With an estimated 2.2 million new cancer cases and 1.8 million deaths, lung cancer (LC) is the second most commonly diagnosed cancer and the leading cause of cancer death in 2020. In men, it is still the most frequently occurring cancer. Lung cancer remained the leading cause of cancer death, with an estimated 1.8 million deaths (18%) [1]. Non-small cell lung cancer (NSCLC) constitutes about 85% of all lung cancer. Its main subtypes include adenocarcinoma, squamous cell carcinoma and large cell carcinoma [2]. Approximately 20–25% of NSCLC patients are candidates for surgical resection with curative intent [2]. However, many patients are at risk of lung cancer recurrence even after complete resection. The 5-year survival rate in resected NSCLC patients has been reported to be over 70% in patients with stage I, to less than 30% in patients with stage IIIA [3]. A high proportion of patients with resected NSCLC die of recurrent disease, suggesting that many of them have micrometastatic disease at the time of surgical resection. Owing to advances in epigenomic and cellular profiling of lung cancer, we have a better understanding of carcinogenesis. This knowledge has led to the development of effective targeted therapies and immunotherapies with survival benefits in selected subgroups [4]. In order to improve the outcomes in early lung cancer, systemic treatment is implemented in the treatment at different time points. Neoadjuvant therapy refers to medicines that are administered before surgery, whereas adjuvant therapy refers to post-surgery treatment. In the era of chemotherapy, there is a choice concerning one of them; immunotherapy, being much less toxic, might be considered as preoperative treatment as well. There is wide discussion on how to implement innovative treatment into early-stage settings [5].

## 2. Chemotherapy in Early Lung Cancer

In an attempt to improve the survival of early-stage NSCLC patients, many trials have been conducted and some have demonstrated the benefits of adjuvant chemotherapy. The Lung Adjuvant Cisplatin Evaluation (LACE) meta-analysis covered the five largest trials evaluating adjuvant cisplatin-based chemotherapy. The LACE meta-analysis included 4584 patients and had a median follow-up of 5.2 years. The results demonstrated a 5.4% increase in the percentage of patients with more than 5 years of survival with a hazard ratio (HR) of death at 0.89 (95% CI: 0.82–0.96, *p* = 0.005) in favor of chemotherapy compared to placebo [6].

Compared to the number of data for adjuvant chemotherapy, data on neoadjuvant chemotherapy are limited. However, there are potential advantages of neoadjuvant chemotherapy including improved tolerability, possible downstaging, and earlier treatment of micrometastases. Neoadjuvant chemotherapy could be considered, especially when the feasibility of surgery is unsettled. However, there are studies suggesting higher perioperative mortality in patients undergoing pre-operative chemotherapy [7].

Although neoadjuvant chemotherapy can lower the risk of relapse, it only provides a pathological complete response (pCR; no viable tumor cells) in 4% of patients. Nevertheless, the five-year survival rate significantly improved in patients with pCR [8].

With wide use of screening programs based on computed tomography examination, the proportion of early-stage NSCLC patients is increasing [9]. Since the benefits of adjuvant and neoadjuvant chemotherapy in early-stage NSCLC patients remain low, there is a need to find more efficient perioperative systemic treatment. Upfront treatment with immunotherapy is a novel perspective.

With the rapid progress made in cancer immunotherapy in patients with metastatic disease, there has been increasing interest in applying immune checkpoint blockade in the neoadjuvant setting for earlier stage malignancies. A rational approach to improve survival in these patients is to eradicate micrometastatic disease and potentially induce anti-tumor immunity to minimize the risk of relapse with peri-operative regimens. The prime advantage is that efficacy of treatment can be assessed preoperatively by sampling tumor tissue and postoperatively by pathologic examination of the resected tumor. Preclinical studies suggest that neoadjuvant immune checkpoint blockade may not only improve surgical ability of high-risk or borderline resectable lesions, but also patients’ survival by decreasing rate of recurrence.

## 3. Neoadjuvant Immunotherapy in Clinical Trials for NSCLC Patients

### 3.1. NEOSTAR

NEOSTAR was the first significant clinical trial with neoadjuvant chemotherapy in patients with surgically resected tumors. It was a phase II trial conducted by researchers at The University of Texas MD Anderson Cancer Center. This randomized study included two arms of patients with early-stage resectable NSCLC. The first arm received nivolumab as single agent, the second arm received a combination of nivolumab and ipilimumab. Both regimens were delivered for three cycles and followed by surgery. The trial enrolled 44 patients who were randomly assigned to both arms [10]. 38% of patients treated with nivolumab plus ipilimumab had a major pathologic response. Whereas, 22% of patients who received nivolumab alone achieved a major pathologic response. The overall major pathologic response rate across both trial arms was 24%. Taking into account only resected tumors (37 patients), the MPR rates were even higher (24% (5/21) and 50% (8/16) of patients treated with nivolumab and nivolumab plus ipilimumab, respectively) (Table 1). High percentages of tumor cells with expression of PD-L1 (programmed death ligand 1) prior therapy was positively correlated with radiographic responses and with pathologic tumor responses at the time of surgery. Compared with nivolumab, nivolumab plus ipilimumab resulted in higher pathologic complete response rates (10% versus 38%), less viable tumor (median 50% versus 9%), and greater frequencies of effector, tissue-resident memory T cells [11].

Additional NEOSTAR endpoints included treatment failure (TFs) rates and disease control rates (DCRs). After a median follow-up of 35 months, TF was observed in 27% of patients, of which 42% of patients had not undergone surgery. Twenty percent of patients included in the study had relapses. Treatment failure was less frequent in smokers (HR = 0.20, *p* = 0.007). Most of the patients who relapsed had genetic aberrations (8/9 patients, 89%), the most common of which was a mutation in the *EGFR* gene [12].

### 3.2. LCMC3

LCMC3 was a multicenter trial exploring the use of neoadjuvant treatment with atezolizumab. Patients with resectable NSCLC received 2 cycles of atezolizumab, then underwent surgery. The treatment also included 12 months of atezolizumab post-resection therapy. Tumor and lymph node biopsies were obtained before systemic treatment and during surgery for biomarker assessment. Neoadjuvant monotherapy with atezolizumab led to a major pathologic response in 19% of patients, as well as a pathologic complete response in 5% of patients (Table 1). In general, presence of PD-L1 expression on tumor cells was significantly associated with response. However, there were patients with major pathologic responses whose tumors were negative for PD-L1 expression. Tumor mutation burden (TMB) analysis revealed that median TMB was 10.4 (range: 1.5–46.5) mutations per Mb and was not different in patients with MPR compared with patients without MPR. In summary, the study failed to identify strong biomarkers of response to immunotherapy [13].

### 3.3. NEOMUN

NEOMUN study is designed to assess the antitumor activity of a neoadjuvant pembrolizumab. It is a single arm, prospective, phase II, ongoing study including patients with NSCLC stage II and IIIA suitable for curative intent surgery. After two cycles of immunotherapy, tumor resection is performed. Except the disease-free rate and overall survival (OS), the study analyses potential predictive biomarkers as well as clinical and pathological tumor response. Although the study will include a modest number of patients, it will cover detailed information of tumor characteristics. This will include the tumor microenvironment, tumor mutational burden, mutational status, other genomic alterations, and cytokine expression levels [14].

## 4. Combination of Immunotherapy and Chemotherapy in Neoadjuvant Treatment in NSCLC Patients

The combination of immune checkpoints inhibitors (ICIs) and chemotherapy may also offer synergistic activity, given that chemotherapy results in tumor cell death and subsequent antigen release that can activate an immune response. Therefore, combining cytotoxic chemotherapy with a PD-1 inhibitor may augment the antitumor response.

### 4.1. NADIM

The NADIM study was a phase II, single-arm, open-label multicenter study aimed to assess the efficacy of combined neoadjuvant chemotherapy and immunotherapy. The study group consisted of lung cancer patients with stage III A disease. Patients were assigned to receive three cycles of neoadjuvant treatment with nivolumab plus chemotherapy with paclitaxel and carboplatin every 3 weeks, followed by adjuvant nivolumab for 1 year. The overall response rate according to radiological criteria was 70% (21 of 30 patients) and included three complete responses (10%) and 18 partial responses (60%). Among the 41 patients who underwent resection, 83% achieved major pathologic response, and 17% had less than 10% of residual viable tumor tissue. The rate of MPR in this study was quite high, particularly in patients with stage III A NSCLC [15,16].

### 4.2. CheckMate 816

CheckMate 816 is an ongoing phase III study evaluating nivolumab plus ipilimumab, nivolumab plus platinum-doublet chemotherapy, and platinum-doublet chemotherapy as neoadjuvant treatment for early-stage NSCLC. This is the largest study with neoadjuvant therapy, and it is planning to enroll approximately 642 patients with early-stage (stages IB-IIIA) resectable NSCLC. The study will focus on event-free survival and pCR rates, as well as overall survival and MPR (Table 1). The estimated primary completion date is May 2023; however, the early results regarding surgical outcomes are promising [17]. Definitive surgery rates were 83% in patients treated with nivolumab plus chemotherapy vs. 75% in patients who received chemotherapy alone. Reasons for cancelled surgery were disease progression, patients’ refusal, unresectability, and decreased lung function. It is important to note that adverse events (AEs) were responsible for delays of surgery in six patients in the nivolumab plus chemotherapy arm, and in nine patients in the chemotherapy arm. Although R0 resection was achieved in the same percentage of patients from both groups, the median residual viable tumor (RVT) cells in the primary tumor bed were 10% in patients treated with combination therapy vs. 74% in patients who received chemotherapy alone. The data from CheckMate 816 support nivolumab plus chemotherapy as a potential neoadjuvant option for patients with stage IB to IIIA resectable NSCLC [18].

### 4.3. IMpower 030

The combination of atezolizumab and chemotherapy demonstrated significant activity in the neoadjuvant setting in the phase II study. Treatment response was seen regardless of PD-L1 score [19]. The results were encouraging enough to move forward and implement a phase III study. IMpower 030 is an ongoing study designed for patients with stage II to IIIB NSCLC eligible for resection with curative intent. Patients are randomized to receive four cycles of neoadjuvant atezolizumab or placebo in combination with chemotherapy chosen by the investigator, followed by adjuvant atezolizumab treatment for 16 cycles. Patients from the control arm receive the best supportive care after surgery and are subjected to observation. Major pathological response (10% residual viable tumor tissue at the time of resection) is proposed as one of the endpoints, together with overall survival and disease-free survival. The study also focuses on biomarkers [20].

### 4.4. CheckMate 77T

CheckMate 77T is the newest phase III trial assessing a combination of chemotherapy and immunotherapy in the neoadjuvant setting for NSCLC patients. The study is designed to enroll over 450 patients with resectable stage IIA–IIIB NSCLC. Patients will be randomized to receive neoadjuvant nivolumab plus platinum-based doublet chemotherapy followed by surgery and adjuvant nivolumab. Study endpoints include OS, pathological complete response, and MPR. The estimated time of results is May 2023 [21].

### 4.5. AEGAN

The AEGEAN study is constructed to assess the activity and long-term clinical outcomes of durvalumab in combination with chemotherapy prior to surgery, as well as further administration of durvalumab. This is a phase III, randomized study which is focused on the efficacy of neoadjuvant combinations in terms of major pathological response [22].

## 5. Adjuvant Immunotherapy in NSCLC Patients

### 5.1. IMpower010

IMpower010 is a phase III, global, multicenter, open-label, randomized study evaluating the efficacy and safety of adjuvant atezolizumab compared with best supportive care (BSC) in NSCLC patients in stage IB-IIIA. All patients after surgical resection received up to four cycles of adjuvant cisplatin-based chemotherapy. The study randomized more than 1000 patients with a ratio of 1:1 to receive up to 16 cycles of atezolizumab or BSC. The IMpower010 study showed for the first time that treatment with atezolizumab following surgery and chemotherapy reduced the risk of disease recurrence or death. Immunotherapy reduced the risk of disease relapse by 34% (HR = 0.66, 95% CI: 0.50–0.88) in stage II-IIIA NSCLC patients with expression of PD-L1 on ≥1% of tumor cells compared with BSC. In all randomized stage II-IIIA study participants, regardless of PD- L expression, atezolizumab reduced the risk of disease recurrence or death by 21% (HR = 0.79, 95% CI: 0.64–0.96) after a median follow-up of 32.2 months. In this population, atezolizumab compared to BSC increased median DFS by seven months (42.3 months versus 35.3 months) (Table 1). Although the addition of up to one year of immunotherapy following chemotherapy led to a higher number of AEs compared with BSC, safety data in this study were consistent with the known safety profile of atezolizumab and no new safety signals were identified [23].

### 5.2. NADIM-ADJUVANT

The NADIM study is aimed at evaluating safety and efficacy of immunotherapy in the adjuvant setting in completely resected, stage IB-IIIA NSCLC patients. This study is ongoing, an open-labeled, randomized, two-arm, phase III, multicenter clinical trial. Patients in the experimental arm receive nivolumab at a dose of 360 mg plus paclitaxel at a dose of 200 mg/m^2^ plus carboplatin at a dose of AUC5 for 4 cycles every 21 days (+/- 3 days). Maintenance adjuvant treatment includes 6 cycles of nivolumab at a dose of 480 mg every 4 weeks (+/- 3 days). Patients randomized to the control arm will receive chemotherapy alone. The primary objective is to evaluate DFS, MPR and pCR (Table 1) [24].

## 6. Predictive Biomarkers for Adjuvant and Neoadjuvant Immunotherapies

### 6.1. Pathological Outcomes

First of all, the standardized definition of MPR and pCR is necessary to use it consistently in clinical trials with immunotherapy. Correlation of MPR and pCR with DFS and OS in these trials will help to determine if MPR or pCR predicts survival. To understand the mechanism of tumor resistance, it is crucial to examine not only pre-surgery specimens but also residual tumors [25].

However, there still are challenges with using this metric for immunotherapy efficacy assessment. First, it is not considered a validated surrogate endpoint in clinical trials and, therefore, it is not currently used for drug approvals. Moreover, the optimal cut point may differ by histology, such as being different for adenocarcinoma and squamous cell carcinoma. This has potential implications for using this in trials that enroll patients of both histological types. Finally, there are some emerging data that MPR may have different value after immunotherapy than after chemotherapy. MPR and pCR measures are yet to prove a direct link to prolongation of overall survival. The pCR indicates that there are no cancer cells after the surgery. It seems to be easier to define pCR than MPR for a pathologist [26,27]. MPR is relatively more challenging, because it is described by the presence of some remaining cancer cells [8]. The pathologist experience might be crucial in defining 10% or less of viable cancer cells in the specimen (Table 1 and Table 2). Tumor heterogeneity of the remaining tumor tissue may not reflect the efficacy of neoadjuvant treatment [28]. The important point is that none of the described studies are personalizing neoadjuvant therapy. Patients are not qualified for adjuvant or neoadjuvant immunotherapy based on molecular markers. There is still an unmet need for developing biomarkers for treatment in this area. The biomarkers would allow us to identify which patients have micrometastatic disease and, therefore, are likely to benefit from the neoadjuvant systemic therapy. Moreover, the selection of best regimen could be based on predictive biomarkers depending on the risk of relapse and treatment efficacy [29].

### 6.2. PD-L1 Expression

The PD-L1 expression on neoplastic cells is the best-studied biomarker used in the qualification of patients for immunotherapy. The choice of the type of first-line immunotherapy in patients with advanced NSCLC depends on PD-L1 expression. Pembrolizumab monotherapy is used in patients with PD-L1 expression on more than 50% of tumor cells [30]. In the remaining patients, combination therapy with pembrolizumab and chemotherapy could be used. PD-L1 expression has been assessed in clinical trials with adjuvant or neoadjuvant immunotherapy. However, patients were enrolled in the studies regardless of PD-L1 expression. Some studies have shown that patients with PD-L1 expression on tumor cells may benefit more from such treatment [31,32]. Although PD-L1 expression is not considered as a prognostic factor, it was associated with tumor grading in early lung cancer not treated with neoadjuvant treatment [33].

### 6.3. Circulating Tumor DNA

DNA released from damaged or apoptotic tumor cells present in blood plasma, referred to here as circulating tumor DNA (ctDNA), can be isolated and utilized in planning cancer treatment. Freely circulating DNA analysis is one of the promising biomarkers in qualification to adjuvant or neoadjuvant immunotherapy. Diehl et al. found that the majority of the patients had significantly decreased or absent ctDNA in peripheral blood after surgery. Moreover, all patients with detectable ctDNA after surgery had a relapse, while those without detectable ctDNA remained in remission [34]. In patients with disease progression, there was an increase in ctDNA levels. In contrast, patients in remission of disease had stable or low ctDNA levels. These observations suggested the use of ctDNA to measure early molecular response during neoadjuvant therapy. Additionally, ctDNA levels after surgery can predict which patients are likely to recur as a result of presence of molecular residual disease [35]. This rapid decline of ctDNA levels may be employed to monitor disease recurrence before radiological progression appears [36]. There are several new preclinical methods trying to identify the optimal early cancer detection method. One of them is the surface-enhanced Raman spectroscopy (SERS) immunomagnetic assay, which is able to detect as few as five tumor cells in 5 mL of blood in lung cancer patients [37].

### 6.4. Radiological Response

Due to the challenges associated with assessing radiological response in immunotherapy, there is an urgent clinical need for imaging-derived biomarkers not only in metastatic changes but also in early lung cancer, especially in clinical trials. 

PET/CT has a significant role in TNM staging in early lung carcinoma, due to its ability to differentiate the tumor mass from the surrounding inflammatory reaction. Using PERCIST criteria, described in detail by Osman and Korashi, can change the TNM assessment in up to 40% of bronchogenic cancers [38]. PET/CT examinations could help to assess an early response, also in terms of hyperprogression or pseudoprogression. Lang et al. suggest that standardized response criteria such as PERCIST, analogous to RECIST, may be helpful in that field [39].

An interesting approach has been provided in a study by Nakajima et al. In this study, an association between MPR and radiomic features (RF) in [18F]-fluorodeoxyglucose ([18F]-FDG) PET and standard CT examinations has been obtained. The authors analyzed PET and CT scans at baseline and after ICIs therapy in early-stage NSCLC patients. There was a significant increase in tumor heterogeneity in CT images in NSCLC patients after ICIs therapy with major pathologic response. This association may reflect increased T cell infiltration or tumor necrosis. In contrast, most [18F]-FDG-based RFs did not distinguish MPR vs. non-MPR tumors, although the sample size was limited [40].

## 7. Conclusions

**Figure 1:**
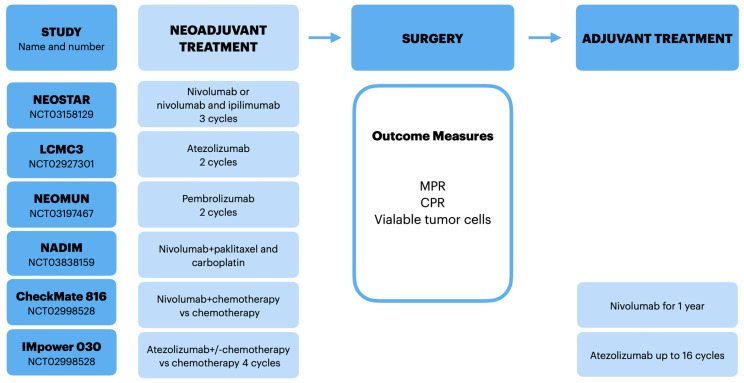
The use of neoadjuvant immunotherapy in NSCLC patients.

In parallel to extending the knowledge in carcinogenesis, new strategies have been implemented in early lung cancer treatment. Some of the studies targeted therapies in patients with genomic alterations either in the advance stage or already registered, e.g., osimertinib based on ADURA trial [41]. A different approach is present in the CANOPY-A and CANOPY N trials where the use of the anti-inflammatory drug canakinumab with or without pembrolizumab is being investigated [42,43]. In this review, we focused on immune checkpoint inhibitors. Neoadjuvant immunotherapy had encouraging activity and demonstrated favorable safety in patients with resectable early-stage non-small cell lung cancer patients. Dissemination of T lymphocytes from the primary tumor may control microscopic metastatic disease. This approach has the potential to improve survival rates in resectable early-stage NSCLC patients according to clinical trials results. Current data, though very limited, suggests that combined immunotherapy is the most active approach. There are several limitations of the use of immune checkpoint inhibitors in neoadjuvant settings. The treatment is better tolerated than chemotherapy; however, immune adverse reaction onset cannot be predicted. Severe pneumonitis, although extremely rare, can deplete the rate of surgical patients. The results of completed studies are encouraging; however, the early phases and small groups should be taken into account. More biomarker research is needed to design personalized treatment strategies. The most effective strategy, adjuvant, neoadjuvant or combined neoadjuvant plus adjuvant immunotherapy regimens, remains unclear. Several clinical studies are dedicated to define the best sequence of treatment (Figure 1).

Adjuvant immune checkpoint inhibitor therapy following neoadjuvant treatment may not be required in most cases. However, much of the important data will be available in the next few years. They will cover the question whether MPR is an adequate surrogate for long-term survival in early-stage NSCLC patients undergoing neoadjuvant immunotherapy. Although major pathologic response and complete pathologic response have been the most commonly used in neoadjuvant trials, the ideal endpoint for neoadjuvant drug approval in NSCLC remains unclear, and there is no consensus whether pCR or mPR is a better endpoint for neoadjuvant trials.

## Figures and Tables

**Table 1:** Results of clinical trials with neoadjuvant therapy in terms of the percentage of patients who achieved MPR and pCR.

Neoadjuvant Immunotherapy Clinical Trials			
Study	Active Treatment	MPR Rates	CPR Rates
NEOSTAR	Nivolumab vs. nivolumab + ipilimumab	24% vs. 50%	10% vs. 38%
LCMC3	Atezolizumab 2 cycles before surgery and 1 year after surgery	19%	5%
Neoadjuvant Chemoimmunotherapy Clinical Trials			
NADIM	Nivolumab + paclitaxel and carboplatin every 3 weeks, followed by adjuvant nivolumab for 1 year	83%	71%
CHECKMATE 816	Nivolumab + 3 cycles of chemotherapy vs. 3 cycles of chemotherapy	36.9% vs. 8.9%	24% vs. 2.2%

**Table 2:** Surrogate measures of efficacy of neoadjuvant treatment.

**MPR**	major pathologic response is currently defined as an “estimated size of viable tumor divided by the size of the tumor bed” of 10% or less
**CPR or pCR**	complete pathologic response was defined as the absence of tumor cells in all resected specimens
**Tumor bed**	applies to the location of the pretreated tumor and includes viable tumor, necrotic areas, and stroma
